# Guillain-Barre Syndrome Deterioration After an Abdominal Surgery

**DOI:** 10.22037/ijcn.v15i3.30815

**Published:** 2022-03-14

**Authors:** Nahid KHOSROSHAHI, Mojdeh HABIBI ZOHAM, Simin KHAYATZADEH KAKHKI

**Affiliations:** 1 Division of Pediatric Neurology, Bahrami Children Hospital, Tehran University of Medical Sciences, Tehran, Iran; 2 Division of Pediatric Intensive Care Unit, Tehran University of Medical Sciences, Tehran, Iran

**Keywords:** Guillain-Barre syndrome, progression, anesthesia, acute abdomen

## Abstract

Guillain-Barre syndrome (GBS) is an acute ascending paralysis accompanied by autonomic symptoms like abdominal pain. Here, we presented a 13-year-old boy suffering from lower extremities pain and sensory disturbances with a presumptive diagnosis of GBS who experienced severe disease progression after receiving general anesthesia due to an appendectomy. Whether the progression was due to the natural history of GBS, immunosuppression induced by surgical stress, or usage of anesthetic medications remained unclear.

## Introduction

*Guillain-Barre syndrome* (GBS) is an acute ascending flaccid paralysis with an incidence of one in 100,000. It involves the lower extremities symmetrically, followed by progression to the upper limbs, face, and intercostal respiratory muscles (1, 2). GBS can affect children of all ages. It is often associated with pain, hyporeflexia or areflexia, ascending weakness, and albumin cytological dissociation in CSF. It can present classically or atypically, including typical (classic) GBS, AIDP (acute inflammatory demyelinating polyneuropathy), AMAN (acute motor axonal neuropathy), or AMSAN (acute motor-sensory axonal neuropathy) (3, 4).

History of illness one to six weeks before the weakness, especially respiratory or gastrointestinal tract infection, might be present. About 30% of children develop cranial nerve involvement (5, 6). Respiratory distress is due to severe upper trunk and diaphragmatic involvement and autonomic disturbances, leading to events such as alterations in blood pressure or tachycardia, which might be life-threatening. Abdominal pain can be the first presenting symptom of autonomic dysfunction (7, 8); however, other etiologies of abdominal pain should be examined first. 

Multiple case reports in adult literature reported GBS occurrence following performing specific surgeries like gastrointestinal, neurosurgical, or procedures that require general anesthesia (9, 10, 11). Whether it is due to a vigorous immunologic response, culminating in the development of polyneuritis, or surgical stress-induced cell-mediated immunosuppression, which in turn might promote infections resulting in the production of cross-reactive antibodies, is unclear (12). To the best of our knowledge, this is the first study in pediatric patients that discussed the progression of GBS following a surgical procedure.

## Case presentation

We reported a previously healthy 13-year-old boy with the chief complaint of lower extremities pain and sensory disturbances since five days ago admitted to Bahrami Pediatrics Hospital, a referral center in Tehran (Iran), in June 2019. The initial evaluation revealed lower limbs weakness and difficulty in sitting and standing up. His past medical history was negative for any significant problem. The patient denied any recent travel or previous gastrointestinal or respiratory infection. His vaccination was updated, with no recent shot. He had no family history of such symptoms and denied using any illicit drug or alcohol.

Physical examination revealed intact consciousness, good cooperation, and orientation to place, time, and person. The vital signs were as follows: blood pressure=119/73 mmHg, PR=88/min, RR=20/min, T=37.6 ͒C, room air oxygen saturation 96%, and body mass index=21 kg/m2. Muscle force in the upper and lower extremities was 5/5 and 2/5, respectively. The gag reflex was present. The absence of DTRs in the lower extremities was evident, with normal DTR of upper extremities. Pupils were normal in size and reactive to light and accommodation. Cranial nerves examination was intact. Other aspects of physical examinations were normal.

Primary investigations were as follows: hemoglobin (Hb) of 12g/dl, total white blood cell count of 9,000 cells/cu mm (N45%, L42%, M5%), random blood sugar of 108 mg/dl, urea of 35 mg/dl, creatinine of 1, and electrolytes, thyroid function tests, liver function tests, and chest x-ray were normal. Also, the toxicology screen test was negative. With a presumptive clinical diagnosis of GBS, electromyography (EMG) and nerve conduction velocity (NCV) were requested, revealing acute demyelinating sensorimotor polyneuropathy.

Thereafter, treatment with IVIG was initiated. On the third day of IVIG treatment, the patient complained of abdominal pain. Abdominal examination revealed tenderness in the right lower quadrant (RLQ) without guarding or rebound tenderness. Abdominal ultrasonography suggested acute appendicitis. Subsequent laboratory results showed a total white blood count of 15,000 cells/cu mm (N 70%, L 22%), hemoglobin (Hb) of 11.8 g/dl, and platelet of 225,000/microliter. Following surgical and anesthetics consultations, an open appendectomy was scheduled. Preoperative measurement of forced vital capacity was not performed due to the unavailability of some equipment. 

The child was transported to the operating room, and following the application of routine monitors, anesthesia was induced with sevoflurane in 100% oxygen and application of midazolam 1 mg IV, fentanyl 70 microgram IV, lidocaine 2% 1cc IV, propofol 70 mg IV, and thiopental IV 125 mg. Following anesthetic induction, the trachea was intubated under deep sevoflurane anesthesia. Anesthesia was maintained with 2% sevoflurane in the air. The patient's vital signs remained stable throughout the procedure. His post-anesthetic course remained unremarkable. Afterward, the patient was transferred to the pediatric intensive care unit (PICU) for meticulous postoperative monitoring.

Two hours after transferring to PICU, he was agitated and experienced shallow breathing with tachypnea, drooling, increased perspiration, and hypertension. Vital signs were as follows: BP=155/75 mm Hg, PR=103/min, RR=32, T=37.2 °C, air room saturation O2=84%, and re breather O2 mask= 93%.

In his secondary neurologic examination, muscle force in the upper and lower extremities was decreased to 1/5. The gag reflex was absent. The absence of DTRs in the lower and upper extremities was evident. Other cranial nerve findings were unremarkable.

Upcoming chest X-ray revealed a new consolidation in the right lung's lower lobe, suggestive of aspiration pneumonia. Appropriate antibiotic therapy (ceftriaxone and clindamycin) was initiated. Continuous infusion of labetalol (0.5 mg/kg/hr) and IVIG drip (400 mg/kg/per day for five days) were administered.

Several hours later, due to deterioration of respiratory problems noninvasive ventilation (NIV, BiPAP, PSV, IPAP=12, EPAP=5, RR=18, and FIo2=90%) was tried for him. Although the patient could properly tolerate NIV for two hours, but we decided to perform endotracheal intubation due to excessive oral secretion and increment in PaCo2 level and subsequent respiratory acidosis in arterial blood gas (ABG). He tolerated intubation with 1 mcg/kg/min of midazolam drip while responding to verbal commands.

Power and tone of both upper and lower extremities were gradually improved following receiving treatment. Weaning trials from the ventilator were carried out, and after nine days, he was extubated uneventfully. Autonomic changes disappeared a few days after initiation of IVIG. The surgical pathology report also confirmed acute appendicitis. The patient was transferred to the neurology ward from PICU and discharged home on hospital day 20.

In 3-months follow-up, minimal residual weakness presented in his extremities; however, DTRs were not detected, and in six months, weakness was resolved absolutely, and deep tendon reflexes reappeared.

## Discussion

Guillain-Barre syndrome (GBS) is the most common immune-mediated peripheral polyradiculopathy. GBS association with infectious triggers (Campylobacter jejuni, CMV, EBV, and Mycoplasma pneumoniae), following vaccination (flu or rabies), or even traumatic events, like a surgical procedure, have been well described (13, 14, 15, 16). The inciting events trigger an antibody response with resultant multifocal demyelination and secondary axonal degeneration (14). Surgery-induced GBS has been reported to occur in 5-9% of adults. The presence of active malignancy or autoimmune process turned out to be more common in patients with post-surgical GBS. Both general anesthesia and conscious sedation procedures might trigger the process of peripheral radiculopathy (12).

In the pediatric population, performing procedures such as MRI might necessitate general or local anesthesia during the course of GBS. Less commonly, unrelated surgical emergencies, as in our case, may lead to an operation. Preoperative bedside measurement of forced vital capacity (FVC) may help the anesthesiologist estimate the risk of impending respiratory insufficiency during the procedure or in the postoperative course (13).

A few adult cases have been described as exacerbating GBS following a surgical process (17). While authorities have suggested the possible role of anesthetic medications or natural history of GBS, strong evidence are not available. Another unproven hypothesis is surgery-induced immunosuppression, leading to progressive axonal degeneration. To the best of our knowledge, this is the first case report in the pediatric population describing the progression of GBS following an operative procedure. 

Furthermore, peripheral neuropathy presenting as an acute onset abdominal pain has been reported in the literature. As an autonomic component of GBS, the authors suggested that abdominal pain should be in the differential diagnosis of the acute abdomen after excluding other etiologies. Moreover, other peripheral neuropathies, such as acute porphyria (axonal polyneuropathy), poisoning with leads or arsenic, may also cause acute abdominal pain (18).

In conclusion, pediatric neurologists and anesthesiologists should be aware of the possibility of exacerbation of GBS patients following procedures requiring anesthesia. Meticulous pre- and post-procedural cautions should be taken to timely diagnosis and provide necessary interventions to improve patients' condition. Prospective, more extensive studies are needed to evaluate the real burden of surgery-induced GBS or exacerbation following a surgical procedure in the pediatric population.

## Author’s contribution

Khosroshahi supervised and managed the patient and approved the final manuscript.

Khayatzadeh Kakhki wrote the first draft of the manuscript and corresponding author

Habibi managed the patient in PICU and edited the paper.

## Conflict of interest

None to declare.
